# A Systematic Review of Sensing Technologies for Wearable Sleep Staging

**DOI:** 10.3390/s21051562

**Published:** 2021-02-24

**Authors:** Syed Anas Imtiaz

**Affiliations:** Wearable Technologies Lab, Imperial College London, London SW7 2AZ, UK; anas.imtiaz@imperial.ac.uk

**Keywords:** sleep, wearables, sleep staging, sleep sensors, sleep scoring

## Abstract

Designing wearable systems for sleep detection and staging is extremely challenging due to the numerous constraints associated with sensing, usability, accuracy, and regulatory requirements. Several researchers have explored the use of signals from a subset of sensors that are used in polysomnography (PSG), whereas others have demonstrated the feasibility of using alternative sensing modalities. In this paper, a systematic review of the different sensing modalities that have been used for wearable sleep staging is presented. Based on a review of 90 papers, 13 different sensing modalities are identified. Each sensing modality is explored to identify signals that can be obtained from it, the sleep stages that can be reliably identified, the classification accuracy of systems and methods using the sensing modality, as well as the usability constraints of the sensor in a wearable system. It concludes that the two most common sensing modalities in use are those based on electroencephalography (EEG) and photoplethysmography (PPG). EEG-based systems are the most accurate, with EEG being the only sensing modality capable of identifying all the stages of sleep. PPG-based systems are much simpler to use and better suited for wearable monitoring but are unable to identify all the sleep stages.

## 1. Introduction

It is estimated that more than 3.5 million people in the United Kingdom and more than 70 million people in United States suffer from some sort of sleep disorder [1,2]. Apart from having a major impact on the quality of lives of individuals, sleep disorders also result in a huge financial impact on the economy stemming from expensive treatments, reduced productivity, road accidents, and many other areas that involve alertness and quick judgement [3,4]. Diagnosis of sleep disorders is an expensive procedure that requires performing a sleep study known as polysomnography (PSG) to monitor multiple parameters and physiological signals during sleep [5]. These may include neural activity (EEG), eye movements (EOG), muscle activity (EMG), heart rhythms (ECG), and breathing functions. Together these signals, or a combination of them, are used to perform sleep scoring and to identify, or rule out, the presence of multiple sleep disorders.

Polysomnography is usually performed at sleep clinics in the presence of trained technicians, typically using four to six EEG electrodes; two EOG electrodes; four EMG electrodes; two ECG electrodes; and additional sensors such as pulse oximeter, sound probes, etc. The signals obtained from these sensors are analysed and scored into one of the different stages of sleep. Human sleep is broadly classified into into Wake, REM (rapid eye movement), and NREM (non-rapid eye movement) stages [6]. The NREM stage is further divided into N1, N2, and N3 stages (or S1, S3, S3, and S4 based on the older Rechtschaffen and Kales (R&K) rules for classification) [7]. The process of assigning a sleep stage to each block of 30-second PSG signal is known as *sleep staging* or *sleep scoring*.

Despite the importance of PSG for the diagnosis of sleep disorders, its high cost coupled with the necessity of clinical admission and long waiting lists [8] limits its usage outside of specialised clinics. Additionally, manual analysis and scoring of sleep from PSG traces is a tedious task that can take 2–4 h for scoring data from an entire night sleep [9]. The advent of low-power wearable sensors and edge computing has resulted in several systems being developed to make PSG easier and to make it possible to perform sleep monitoring with automatic sleep detection and staging over multiple nights at the home of patients [10]. Researchers have used several sensors to obtain physiological parameters during sleep and developed various methods for their processing to classify them into sleep stages. Two intrinsically linked areas of research have emerged, with one focusing on signal acquisition and the other on signal processing. In the case of signal acquisition, there is a push towards finding alternative sources of sleep information than those traditionally used for PSG. This involves developing new sensors, interfacing circuitry, inplementing low-power hardware, as well as integrating everything into smaller packages. The signal processing research focuses heavily on using techniques to perform feature extraction and on taking advantage of the advances in machine learning for classification.

While several review papers have focused on the algorithmic accuracy of different methods for automatic sleep detection and staging using a variety of physiological signals [11], this paper presents a systematic review of the sensing technologies available to acquire those signals. It focuses on systems and methods that are useful for long-term sleep monitoring through the use of home-based wearable devices. It presents a comprehensive discussion of the relevant sensing technologies focused on their suitability, limitations, usability, as well as their ability to extract signals that can reliably be used for automatic sleep detection and staging. The rest of this paper is organised as follows. Section 2 describes the review methodology used to identify candidate papers for this review. Section 3 synthesises the results presenting an overview of the different sensing modalities that are used for wearable sleep detection and staging. For each sensing modality, the challenges associated with signal acquisition, reliability, usability, and accuracy are discussed as well as a review of the different papers that have used the sensing modality in the context of automatic sleep staging. Finally, Section 4 discusses the overall landscape of sensing technologies looking at the different trade-offs that need to be taken into account when designing a wearable device for sleep detection and staging.

## 2. Methodology

### 2.1. Data Sources

**Figure 1:**
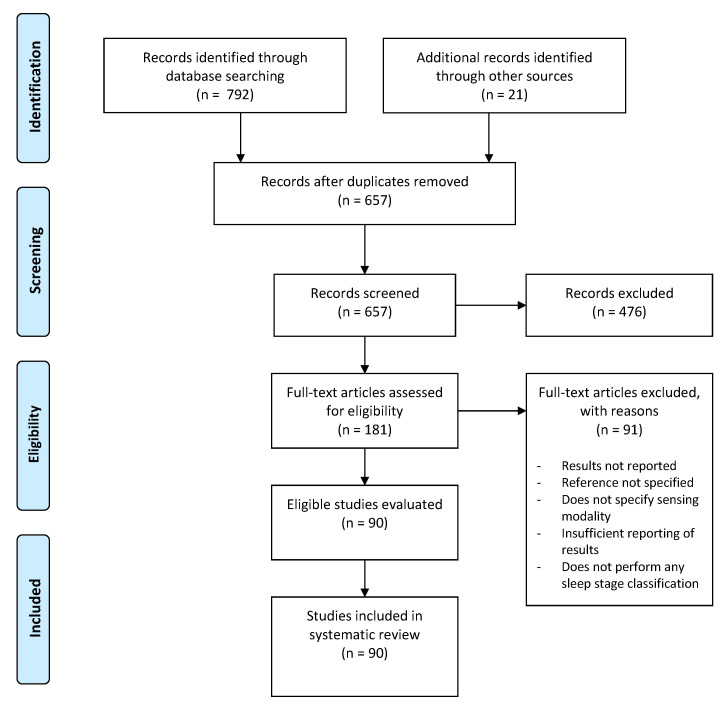
Study selection flow diagram.

This systematic review is performed using the guidelines of the Preferred Reporting Items for Systematic Reviews and meta-Analysis (PRISMA) statement [12]. It includes peer-reviewed articles written in English language published between 2010 and 2021. The lower limit of year 2010 is used because of the more recent developments in the area of wearable computing and systems. The initial list of articles was compiled by searching on the PubMed, SCOPUS, and IEEEXplore databases. The electronic search terms used with PubMed (as well as other databases) included wearable sleep staging, wearable sleep scoring, wearable sleep, wearable sleep eeg, wearable sleep ecg, wearable sleep sensors, wearable sleep breathing, wearable sleep plethysmography, wearable sleep ppg, wearable sleep actigraphy, and wearable sleep movement. The search made use of the binary OR operator to look for the presence of these terms in the title, abstract, or keywords of the articles and limited the publication year to be at least 2010. Additional articles were obtained from the bibliographies of articles found. The date of the last search was 8 February 2021. The initial search resulted in 813 articles that were further screened to remove duplicates and assessed against the eligibility criteria described in the next section. A flow diagram for identification, screening, and eligibility of the papers to be included in the review is shown in Figure 1.

### 2.2. Eligibility Criteria

The initial set of articles were filtered to remove the duplicates and to narrow down the scope in order to include only articles that were focused on designing a system or algorithm for wearable sleep detection and staging. This may include either a sensor designed specifically for sleep staging, including distinction between sleep and wake, or a method developed using data from a sensing source that could be used in a wearable context. This resulted in 181 articles that were deemed relevant. This number was further reduced to 90 after the full-text was assessed against the following eligibility criteria.

- Does the article present a sensor/method for sleep staging?
- Does the sensor/method in article classify at least one stage of sleep (including wake as it is considered a distinct stage based on both the R&K and AASM rules of classification)?
- Is the sensor/method for use for staging human sleep?
- Does the article discuss potential use of the sensor/method in wearable context? If not, is the sensor/method obviously suitable for wearable use (e.g., wrist-based devices)?
- Is the sensor/method designed for monitoring overnight sleep (i.e., not only daytime naps)?
- Is the sensing modality clearly defined?
- Has the work being presented been validated against a reference method?
- Does the study report at least one quantifiable measure of accuracy?

### 2.3. Data Extraction

Data from each study was extracted into a spreadsheet that had columns for all the variables sought for this review. Where relevant, accuracy measures were computed from the reported metrics in the paper and entered into this spreadsheet. From each study presenting a system or method for automatic sleep staging, data were sought on the sensing modality of the system used for data collection, the classification accuracy of the method, the sleep stages being classified, the validation reference, the location of data collection, the age range of subjects, population size, and the source of data (i.e., an actual device or an existing database).

### 2.4. Risk of Bias

There is a risk of bias in individual studies reporting results based on different validation references. Even when the validation reference is the same, manual scoring by sleep technicians is subjective and hence may have some influence on the reported results. Additionally, sample size, study location, stages classified, and the reporting metrics themselves can also impact the results. To minimise these risks, two well-known measures of overall accuracy have been used and only those studies that report at least one of these measures were included. Furthermore, the other variables that may have an impact are clearly reported in the results.

Overall, the studies that have been included focus on systems and methods designed for use in wearable sleep systems. Many studies do not mention the wearable or ambulatory nature of devices. Hence, an assessment was made for each study (with insufficient context) to determine its eligibility against a subjective criterion of its possible use in a wearable context.

## 3. Results

**Figure 2:**
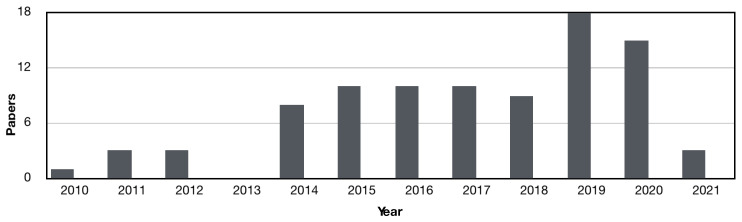
Number of papers published each year since 2010 related to the topic of wearable sleep staging.

Figure 2 shows the frequency of articles published each year. The trend is very clear, with the number of published articles increasing from 2014 onwards, with a small number of articles before that time. This is not surprising since ultra-low power processors and miniaturised electronics have become readily available in the last few years, leading to an exponential increase in research in the area of ambulatory and wearable devices. Additionally, there has also been an increased focus on sleep and its importance for overall health and wellness, leading to a rise in wearable devices being developed to monitor sleep.

Table 1 lists the eligible articles included in this review, summarising their sensing modalities, extracted signals, and sleep stages classification. The different sensing modalities together with their usability, accuracy, and ability to classify different sleep stages are discussed in the following sections.

### 3.1. Overview of Different Sensing Modalities for Sleep Staging

The gold standard for sleep staging is polysomnography (PSG), which involves monitoring of brain waves, eye movements, and muscle movements amongst other physiological parameters. However, due to the difficulties in using PSG outside of clinics, alternative sensing modalities have been proposed for extracting information related to the different stages of sleep. Table 1 lists 13 different sensing modalities that have been used by researchers to extract various signals for sleep detection and staging. Before discussing these sensing modalities, a brief overview of their usage for sleep detection and staging is presented.

#### 3.1.1. Electroencephalogram

An electroencephalogram (EEG) is a recording of the electrical signals of the brain. It is performed by placing several electrodes on different locations of the scalp. The electrodes then feed the signals into a front-end electronic system consisting of amplifiers, filters, and other data acquisition circuitry. EEG signals are highly useful to assess the health of the brain as well as the diagnosis of different sleep and neurological disorders. As part of PSG, for sleep staging, at least three channels of EEG are used to acquire signals from different locations on the scalp. The relative levels of signals at different frequency bands (e.g., alpha (8–13 Hz), delta (0.5–4 Hz), etc.) are then used to identify the sleep stages following the guidelines of the American Academy of Sleep Medicine (AASM) [6]. Although EEG is the signal with the richest information related to sleep, it is difficult to use due to multiple issues such as electrode displacement and noise [133,134,135].

#### 3.1.2. Electrooculogram

An electrooculogram (EOG) is a signal that is generated as a result of eye movements and captured using electrodes placed near the eyes. It is highly useful to identify the wake and REM stages of sleep since there are major eye movements during these stages. Generally speaking, eye movements tend to slow down with the depth of sleep.

#### 3.1.3. Electromyogram

An electromyogram (EMG) is a recording of the electrical signals generated as a result of muscle movements. Electrodes placed on specific muscles result in changes in the signal level whenever those muscles move. As part of full PSG, leg and chin muscle movements are recorded through EMG. Leg movement activity is useful for the diagnosis of specific sleep disorders such as periodic limb movement disorder. Chin movement activity helps to differentiate wake and REM sleep stages from those with similar EEG characteristics.

#### 3.1.4. Electrocardiogram

An electrocardiogram (ECG) is a recording of the electrical activity of the heart. It provides a snapshot of the regular functioning of the heart as well as information about the heart rate. ECG is not conventionally used for sleep staging and is not a required signal for scoring sleep based on AASM guidelines [6]. However, researchers have shown that different features extracted from ECG such as R-R intervals, heart rate, and heart rate variability (HRV) correlate with the changes in sleep macrostructure and thus can be useful for identifying the different stages of sleep [136].

#### 3.1.5. Photoplethysmogram

A photoplethysmogram (PPG) is a signal representing the changes in blood volume in the microvascular bed of tissue [137]. It is obtained by using a simple optical measuring technique where an LED is used to shine light on the tissues and a photodetetor is used to absorb the reflected light. The PPG can then be used to measure the heart rate, resipiratory rate, and oxygen saturation levels. In clinical practice, PPG-based pulse oximetry is popularly used for continuous measurement of oxygen saturation. However, it suffers from reliability issues mainly due to artefacts emanating from movement of the finger. More recently, wearable PPG sensors have been incorporated in wrist-worn consumer devices to extract the heart rate, making it widely accessible [95,138]. This is useful for sleep monitoring since researchers have shown that the changes in heart rate and respiratory rate can be useful for identifying the different stages of sleep [139,140].

#### 3.1.6. Accelerometer

Accelerometers are inexpensive and easy-to-use sensors that record periods of movement and inactivity during sleep and can be used to assess the sleep/wake cycles. They are used for long-term sleep monitoring through wrist-worn devices, also known as actigraphy [141].

#### 3.1.7. Respiratory Inductance Plethysmography

Respiratory inductance plethysmography (RIP) is a noninvasive tool to monitor breathing patterns. It uses a belt placed around the abdomen (or thorax) and measures abdominal movements that are correlated with respiratory movements during inspiration and expiration. Since the respiratory effort changes throughout sleep [142], the RIP signal can be useful for studying and identifying different stages of sleep.

#### 3.1.8. Pressure Sensors

Some researchers have developed sensitive pressure sensors installed on a mattress to record body motion and ballistocardiography (BCG) during sleep. These sensors are used to extract information such as respiratory rate, heart rate, and movements in bed [143,144]. These can be useful for identifying different sleep stages since the body movement is reduced in deep sleep whereas respiratory rate changes are also observed compared to light sleep or wake stages.

#### 3.1.9. Radar Sensors

Radar sensors are used as a non-contact and wireless method of breathing and movement detection [145]. These signals can then be used for sleep staging.

#### 3.1.10. Audio

Microphones placed in various configurations are used to record audio signals during sleep. These signals capture the changes in breathing sound intensity, snoring, and other sounds due to abrupt movements that can be used for sleep staging [146].

#### 3.1.11. Nasal Airflow

Thermal airflow sensors (thermistors or transducers) are used to measure airflow through the nose. The nasal airflow signal shows changes in breathing, which can be helpful for identifying sleep stages [147].

#### 3.1.12. Sonar Sensors

Similar to radar sensors, sonar sensors can also be used as a non-contact and wireless method of breathing and movement detection and subsequently for detecting different sleep stages.

#### 3.1.13. Electrodermal Activity Sensors

Electrodermal Activity (EDA) sensors are used to measure skin conductance at the wrist, palm, or fingers. The EDA levels are known to be higher during deep stages of sleep compared to the lighter ones and hence can be used to differentiate N2 and N3 stages from others [148].

### 3.2. Discussion on Sensing Modalities

The different articles included in this review used at least one or more of the sensing modality described in the previous section. Table 2 shows the number of articles that used each of the sensing modalities in their sleep staging method or system either as the only source of signal information or in combination with other sensors.

Of the 90 articles reviewed, 61 used one of the 13 sensing modalities as the only source of information whereas the others used a combination of two or more of the different sensing modalities. It is not surprising that EEG is the most popular choice of sensing modality when it comes to creating wearable sleep detection and staging systems, where only a single sensing source is used. This is because it is part of the PSG gold standard and the AASM guidelines for sleep scoring are based on the interpretation of EEG signals. Hence, as a signal source, it is considered the one providing the most information that can be clearly used to classify the different stages of sleep while adhering to the medical guidelines. Following EEG, the second most used single-source sensing modality is the accelerometer. This is mainly a consequence of the prevalence of their use in actigraphy, which allows for capturing sleep/wake patterns. The third most used single-source sensing modality is the ECG. This is perhaps due to its ability to provide accurate heart rate and heart rate variability that has been shown to correlate with the different sleep stages [136]. The other sensing modalities are much less commonly used on their own.

Accelerometers, on their own, have been in use for a very long time for actigraphy to detect sleep and wake states. While they do not provide more detailed sleep staging information, they are considered an acceptable and useful method to assess and treat disorders related to sleep patterns [149]. When used in combination with other sensors, accelerometers can be used reliably to refine the sleep staging outputs. For example, when used with EEG, they may be helpful in differentiating between wake and REM stages that have similarities in the EEG and usually require further input from EOG and EMG channels (however, this will not be possible during periods of quiet wakefulness with little to no body movements). Because of their ease of use for patients as well as their low cost of development, accelerometers are the most common sensing modality used in combination with other sensors. The other most commonly used sensing modality in combination with other sensors is the PPG. This is because of the recent explosion in wrist-worn consumer wearables that incorporate PPG sensors providing heart rate information that can be subsequently used for sleep detection and staging. From Table 1, it can be seen that PPG is most commonly used with accelerometers. However, these two sensors together commonly provide 4-state sleep staging rather than full five-state sleep staging. EEG and ECG sensing modalities are also used at times with other sensors despite being quite information rich on their own. In most of the cases where EEG and ECG are used with other sensors, they are the predominant source of information using other sensors only to refine and improve the classification accuracy.

It should be noted though that the use of accelerometers as a sensing modality is highly prevalent for actigraphy studies. They have been in use for a long period of time, are inherently wearable and cost effective, and may be used to target generalised sleep disorders; there are more studies published using such systems. They are also available in millions of consumer devices around the world, making them easier to use for various studies. As a result, it is likely that more studies have been published using these systems, resulting in a higher number of papers based on them.

### 3.3. Signals and Features

When the sensing modality of EEG is used, the signals extracted are brain waves. The main difference between different systems that use EEG is the number of channels and the channel location. For example, single-channel EEG-based systems may prefer frontal channels over the central ones due to the ease of using the electrode. Nevertheless, regardless of which channels are used, the main objective is to interpret their spectral content and to score the signal based on the AASM guidelines. The other sensing modalities, however, acquire physiological signals and process them to score sleep stages despite the lack of any standardised guidelines for those signals. These include cardiac signals, respiratory signals, and body movements.

The different features of cardiac signals that have been used include the heart rate, heart rate variability, and R-R intervals. These may in turn be extracted from different sensing modalities. For example, the heart rate (and pulse rate) is commonly obtained using ECG or PPG. The respiratory feature most commonly extracted is breathing rate. A number of different sensing modalities are used to acquire respiratory signals. These include RIP to measure the effort, audio sensors to record breathing sounds, and non-contact radar sensors and pressure sensors to measure movements, which are subsequently used to extract the breathing rate. Additionally, tri-axial accelerometers have also been used to measure chest movement while breathing to obtain the breathing rate. When placed on wrists, accelerometers are commonly used to obtain general body movement signals that are used in actigraphy.

Of all the articles reviewed in this paper, 19 use signals and features extracted from EEG. Amongst the articles presenting non-EEG-based systems, more than 35 use at least one feature extracted from cardiac signals. This is followed by 24 articles using movement signals and 17 using respiratory features. Thus, it can be concluded that, when using non-EEG sensing modalities, cardiac signal features are the most popular choice for obtaining sleep staging information.

### 3.4. Sleep Stages

The AASM defines five different stages of sleep (wake, N1, N2, N3, and REM), whereas the previous R&K guidelines defined seven stages (wake, S1, S2, S3, S4, and REM). Of all the reviewed articles, only 21 performed classification of all the sleep stages defined by either of these guidelines [24,39,42,45,50,58,67,72,76,80,88,89,90,93,101,102,105,108,109,117,121]. Amongst them, all but three [24,58,150] used EEG signals for classification, where the difference between sleep stages is known to be most obvious. In [150], where only PPG signals are used with accelerometry data to detect movements, the overall accuracy is quite low. This highlights the fact that a clear distinction between different stages is challenging with limited information, which is a consequence of the constraints in a wearable device. It is well known that, even when using EEG signals, the identification of N1 and REM sleep stages are rather challenging [151] and thus require additional sensing modalities in the form of EOG and EMG. Hence, some researchers combine N1 and N2 stages and refer to it as the *light* stage of sleep with N3 (or S3 and S4 combined) referred to as *deep* sleep. Others opt to group sleep into three classes only: wake, NREM, and REM. These approaches are most commonly seen used in systems where the sensing modality is based on PPG, ECG, and radar. Where only accelerometry is used, the only reliable information that can be inferred is the distinction between sleep (no movement) and wake (movement) stages.

Grouping sleep stages together to form a different class helps to improve classification accuracy. However, apart from accelerometry-based actigraphy systems, the usefulness of this approach for medical diagnosis is still to be determined. It is perhaps for this reason that popular wrist-worn PPG-based devices are not regulated for use as medical devices and only marketed as consumer devices.

### 3.5. Accuracy

The accuracy of classification of different sleep stages not only depends on the type of signal obtained from different sensing modalities but also on the algorithms developed to process them further. Direct comparison between the performance of different systems is difficult due to a number of factors [152], including the fact that the metrics used by the authors of different studies can be very different. Further, since there are multiple sleep stages, Cohen’s kappa is a better measure to indicate the accuracy of classification. Consequently, both the reported classification accuracy and kappa values are included in Table 1. It should be noted that these values have been obtained from the papers and that, if they were unavailable, they were computed from the reported results (where sufficient information on classification was available). The studies being performed in different locations with different sample sizes and different stages being classified represent a potential source of bias in the reporting of results. Hence, Table 1 lists the various potential bias sources, and the accuracy needs to be looked at in context. While the effect of study location is not entirely clear, it is likely that those studies classifying smaller number of sleep stages achieve higher accuracy.

Despite the accuracy reporting challenges, it can be noted, with some exceptions, that the majority of of PPG-based three- or four-class systems are able to achieve classification accuracies between 65–75%, with kappa values between 0.5–0.6. This is because some of these systems using PPG and accelerometry together tend to overestimate N3 sleep and to underestimate other stages [153]. Those using EEG as the signal source are able to achieve classification accuracies between 80–90%, with the highest kappa values of over 0.8 indicating strong agreement. If the classification stages are limited to sleep and wake classes, then accelerometers can also achieve 90% accuracy. Additionally, classification based on signals obtained from pressure and radar sensors for the wake, NREM, and REM stages are shown to achieve accuracy up to 80%. In general, systems based on EEG have the highest accuracy and kappa values whereas others based on accelerometers and non-contact sensors have the lowest for 5-state staging [154,155]. While EEG-based methods achieve higher classification compared to other sensing modalities, it should be noted that the studies included in this review with EEG sensing modality predominantly use a single channel or limited number of channels for data acquisition. This is because of the usability challenges associated with multi-channel EEG systems that make them unsuitable for wearable sleep staging. However, while better than other sensing modalities, the accuracy of single-channel EEG systems are reduced when compared to multi-channel sleep staging systems. In particular, a loss in accuracy is more pronounced in the REM and N1 stages, resulting in a decrease in overall agreement [60].

### 3.6. Usability

Wearable devices are a relatively recent development, but several usability studies have already been published to understand the requirements to make them accessible for a wide range of population [156,157]. Although the usability requirements of wearable sleep staging devices are similar to those of other wearables, where different trade-offs pertaining to the battery life, power consumption, human factors, size, and weight need to be taken into account, there are certain additional constraints [158]. For example, since they are likely to be worn just before sleep, they need to be very easy and quick to put on. If this is not the case, user compliance will be very low. Additionally, compared to wearables used during the day, devices designed to monitor sleep need to be more comfortable to avoid disrupting sleep. These are the main reasons why polysomnography is difficult to use on a regular basis outside of clinics without the supervision of trained staff to connect the various electrodes.

The general trend with wearable sleep detection and staging systems is to use a reduced subset of PSG sensors. Most notably, these include using ECG or one-channel EEG that is considerably easier to use for patients and to provide a wealth of information useful for scoring the signals. However, the reliability of signals due to electrode displacement throughout the night remains a challenge [133,134]. Additionally, the cost of EEG-based systems are higher due to the advanced electronic circuitry needed to acquire high-quality signals. As a result, using PPG and accelerometry with wrist-worn devices remain the most usable option in terms of comfort for patients. These sensors are already available in a myriad of consumer devices with integrated software to analyse sleep stages and thus do not interfere with the normal lifestyle of patients. However, due to the inherent sensing limitations, systems using these sensors are unable to identify all five stages of sleep.

### 3.7. Power Consumption

Power consumption is a major design consideration in wearable systems, particularly for sleep staging, where any device should be capable of running for 8–10 h at least, which is the duration for normal sleep. Unfortunately, there are very few articles that have reported developing specific systems and their power consumption. A single-channel EEG-based integrated circuit for automatic sleep staging developed in [72] reported a power consumption of 575 μW. Another integrated circuit using EEG and EMG channels in [101] reported significantly lower consumption at 5 μW. Finally, an integrated system using EEG, EOG, and EMG channels in [150] reported 71 mW as the power consumption of their system. Apart from the latter system, which has a relatively higher power consumption, all are capable of delivering multi-night recordings using small coin cell batteries with a typical capacity of 200–300 mAh.

### 3.8. Challenges and Future Directions

The rise in consumer focus on sleep awareness as well as an increase in understanding the importance of sleep [159] will lead to many more wearable devices being developed to quantify the different sleep stages and to assess sleep quality. Based on the current trends, it is likely that these future systems will become smaller and easier to use for the patients. From a technical development viewpoint, they are likely to use a maximum of one or two sensing modalities to extract direct or indirect parameters that can be mapped to the sleep stages. However, there are a number of usability, reliability, and regulatory challenges that need to be explored.

It is pertinent to note that, regardless of the sensing modality used, sleep staging is only a subset of full polysomnography and, thus, any wearable being developed for sleep staging cannot replace PSG. It can, however, be used to triage patients and to prioritise access to PSG for those who need it the most. Additionally, it can be useful for long-term sleep monitoring of patients as well as other subjects for research. This will require overcoming usability challenges so that patient compliance over several weeks is high. This, in turn, requires creating systems that do not inconvenience users by requiring them to charge the devices regularly or to negatively impact their comfort during sleep.

Although an increasing number single-sensor devices are developed with improved accuracy, their utility and acceptability in the medical community is limited. For example, the heart rate is used in most commercial fitness trackers for sleep scoring. This is advantageous since the heart signal can be useful as an indicator for other health conditions. However, there are no standard guidelines that define how heart rate changes should be mapped on to the sleep stages. This is true for all signals other than those obtained from the EEG. As a result, such devices are not regulated as a medical device; thus, their diagnostic utility is currently very limited [155]. Future research should tackle this by carrying out large trials to establish the diagnostic utility of non-EEG-based signals for sleep detection and staging.

More and more wearable sensors are being developed to monitor cardiac, respiratory, and other physiological parameters [160,161,162,163]. Despite the shrinking size of sensing technologies and low-power electronic circuitry, one of the limiting factors that remains in PSG is the process of electrode attachment. This is essentially a usability issue that requires more research in the area of flexible electrodes that can be used reliably over a longer period of time. This is important since a wearable multi-channel PSG will provide more diagnostic value than a wearable single-channel sleep staging system.

Finally, already an important area of focus for researchers, processing and classification of sleep signals will continue to grow. This will involve creating more low-complexity and higher accuracy algorithms for signals acquired from the different sensing modalities. However, algorithms developed for similar signals obtained from different sensing modalities may not be compatible with each other [164]. Further, the limited availability of accessible data sets makes it difficult to develop and benchmark such algorithms [165]. Having a wearable system will make it easier to obtain and maintain large data sets of different sleep signals that could be helpful for researchers to assess and compare the accuracy of their algorithms. Since the scope of this paper is limited to sensing modalities, readers interested in a review of algorithmic approaches for automatic sleep staging are referred to [11]. Other deployment challenges of long-term home-based devices include their management, security, updates, and maintenance, as discussed in [166].

## 4. Conclusions

The limitations of PSG due to its usability and cost has resulted in a number of different sensing modalities being explored for sleep detection and staging. Despite the issues with PSG, it can be concluded that, when a single sensing modality is used, EEG is by far the most popular choice, given that it conforms broadly to the AASM scoring guidelines. There are usability and reliability issues with the use of EEG; however, recent advances in the area of wearable EEG [167] such as ear-EEG and tattoo-like electrodes are likely to help with these and to improve the likelihood of obtaining reliable signals during sleep. When a combination of sensors are used, PPG and accelerometry together are the most popular option. This is partly because these sensors are widely available in convenient wrist-worn consumer devices. However, regardless of the convenience in use, this combination of sensing modality is unable to identify all stages of sleep and its accuracy is also lower compared to EEG. Further, as a result of the low accuracy of commercial systems that use this sensing modality for sleep staging, they are not deemed a suitable alternative to PSG [138]. Thus, it appears that a wearable EEG is more accurate compared to PPG and accelerometry whereas the latter is a more attractive option if usability is more important. Other sensing modalities such as ECG, radar, audio, and pressure sensors are also a more user-friendly option with limitations on what sleep stages they can reliably discern.

Although the results in this paper show a clear trend towards specific sensing modalities, there are certain limitations of this study. For instance, because of the focus on sensing modality, the discussion of the different algorithms and processing requirements is not included. This, however, is important from a system design viewpoint since some of the computational requirements for implementing these algorithms can add additional constraints to the sensing power consumption. Additionally, as with the design of any wearable device, various other factors will have different weights depending on the application and certain trade-offs will be needed. For example, a device designed for medical use will need a higher accuracy with resolution of all the stages of sleep whereas a consumer sleep monitoring device will lean towards usability as the most important factor. Nevertheless, the results presented in this paper can help the designers of wearable sleep detection and staging devices make these informed choices and trade-offs. Understanding the choice of sensor for wearable sleep staging and the limitations on signals obtained from that sensor is still important on its own, and thus, the results of this paper can be useful for designers of wearable devices for sleep detection and staging to make important decisions at early stages of their product development life cycle.

## Figures and Tables

**Table 1:** Summary of articles included in the review.

Ref	Sensing Modality	Signals/Features	Sleep Stages Classified	Accuracy	Kappa	Validation	Subjects	Age	Location	Data Source
[13]	Pressure sensors	HBI + Movement	Wake-NREM-REM	79%	0.44	PSG	9	20–54	Hospital	Emfit [14]
[15]	ACC + PPG	HR + Movement	Wake-REM-Light-Deep	65.3%	0.48	PSG	227	36–63	Sleep Lab	WatchPAT 100 [16]
[17]	RIP + Nasal airflow	Respiratory effort	Wake-NREM-REM	70%	-	PSG	16	32–56	Hospital	MIT-BIH [18]
[19]	ACC	Movement	Wake-Sleep	86.2%	0.65	PSG	30	17-24	Research Lab	Actiwatch-64 [20]
[21]	EEG	1 channel	Wake-REM-Light-Deep	81.1%	0.7	PSG	26	19–60	Sleep Lab	Zeo
[22]	PPG + ACC	HRV + Movement	Wake-NREM-REM	75%	-	PSG	48	22–71	Hospital	Research Device
[23]	ECG	HRV	Wake-NREM-REM	73.50%	-	PSG	7 (OSA)	42–68	Hospital	Unknown
[24]	ACC	Hip Movement	Wake-N1-N2-N3-REM	88.4%	-	PSG	34	20–24	Sleep Lab	FS-750
[25]	ACC	Movement	Wake-Sleep	88.0%	0.3	PSG	22	20-28	Sleep Lab	Actiwatch-64 [20]
[26]	RIP + ACC	Effort + Movement	Wake-Sleep	95.7%	0.66	PSG	15	23–58	Sleep Lab	Actiwatch [20]
[27]	RIP + ECG + ACC	RRI + Movement	Wake-NREM-REM	78.3%	0.58	PSG	20	32–52	Sleep Lab	ACT/Somnowatch [28]
[29]	EEG	1 channel	NREM-REM	83%	0.61	PSG	20	20–65	Hospital	DREAMS [30]
[31]	EEG	1 channel	Wake-N1	92.50%	0.63	PSG	13	-	Hospital	Sleep EDF Expanded [32]
[33]	PPG	PRV	Wake-NREM-REM	77–80%	-	PSG	146 (SDB)	9–14	Hospital	Unknown
[34]	Pressure sensors	RR + Movement	Wake-NREM-REM	70.30%	0.45	PSG	7	21–60	-	Research Device
[35]	ACC	Hip Movement	Wake-Sleep	86.2%	-	PSG	108	21–24	Research Lab	GT3X+ [36]
[37]	ACC + EEG	Movement + 1 channel	Wake-REM-Light-Deep	74.2%	-	PSG	30	18–80	Sleep Lab	SomnoScreen [38]
[39]	EEG + RIP	Respiratory effort	Wake-N1-N2-N3-REM	81.70%	-	PSG	-	-	Hospital	Unknown
[40]	ECG	HR	Wake-REM-Light-Deep	76–85%	-	PSG	15	28–39	Hospital	CAP Sleep Database [41]
[42]	EEG	1 channel	Wake-N1-N2-N3-REM	79%	0.69	PSG	39	25–101	Hospital	Sleep EDF Expanded [32]
[43]	EEG	1 channel	Wake-REM-Light-Deep	-	0.72	PSG	99	18–60	Sleep Lab	Zmachine [44]
[45]	EEG	1 channel	Wake-N1-N2-N3-REM	86.20%	0.78	PSG	8	21–35	Hospital	Sleep EDF Database [32]
[46]	Radar sensors	Respiratory movement	Wake-NREM-REM	75%	0.56	PSG	29 (SDB)	22–67	Hospital	Research Device
[47]	RIP	Respiratory effort	Wake-NREM-REM	80%	0.65	PSG	29 (SDB)	22–67	Hospital	Embla N7000 [48]
[49]	Radar sensors	RR + Movement	REM-Light-Deep	79.30%	-	PSG	11	21–23	Research Lab	Research Device
[50]	EEG + EOG + EMG	Ear-EEG	Wake-N1-N2-N3-REM	95%	-	PSG	8	avg. 25	Sleep Lab	Research Device
[51]	Radar sensors	HRV + Movement	Wake-Sleep	66.4%	-	PSG	10	21–24	Research Lab	Research Device
[52]	ECG	RRI	NREM-REM	76.20%	0.52	PSG	20	22–33	Hospital	MASS PSG Database [53]
[54]	ECG + PPG	HR + Oximetry	Wake-Sleep	83.80%	-	PSG	100	-	Home	SHHS Database [55]
[56]	Audio	Respiratory features	Wake-NREM-REM	75%	0.42	PSG	20	23–70	Hospital	EDIROL R-4 [57]
[58]	ECG + PPG	HRV + PTT	Wake-N1-N2-N3-REM	73.40%	-	PSG	20 (Insomnia)	-	Hospital	SOMNOScreen [38]
[59]	Nasal airflow	RR	Wake-NREM-REM	74%	0.49	PSG	20	25–34	Hospital	Sleep EDF Expanded [32]
[60]	EEG	1 channel	Wake-N1-(N2+N3)-REM	90%	0.67	PSG	29	-	Research Lab	Sleep Profiler [61]
[62]	ACC	Movement	Wake-Sleep	-	-	PSG	14	3–11	Hospital	Fitbit Flex [63]
[64]	ACC + PPG	HR + Movement	Wake-Sleep	90.90%	-	PSG	32	14–20	Sleep Lab	Fitbit ChargeHR [63]
[65]	EEG	Ear-EEG	Wake-N1-N2-N3	76.80%	0.64	EEG	4	25–36	Research Lab	Research Device
[66]	ECG + RIP	RRI + Respiratory effort	Wake-REM-Light-Deep	87.40%	0.41	PSG	180	20–95	Hospital	Multiple Databases
[67]	EEG	1 channel	Wake-S1-S2-S3-S4-REM	90.5%	0.8	PSG	20	25–34	Hospital	Sleep EDF Expanded
[68]	PPG + ACC	HRV + Movement	Wake-REM-Light-Deep	69%	0.52	PSG	60	24–44	Home	Fitbit Surge [63]
[69]	ECG + ACC	HR + Movement	Wake-NREM-REM	75%	0.49	PSG	289 (Various)	37–65	Hospital	Unknown
[70]	PPG	HRV	Wake-Sleep	80.10%	-	PSG	-	-	Hospital	Multiple Databases
[71]	ACC + PPG	HRV + Movement	Wake-N1-(N2+N3)-REM	59.3%	0.42	PSG	51	41–60	Home	Alice PDx
[72]	EEG	1 channel	Wake-N1-N2-N3-REM	79%	0.59	PSG	20	20–65	Hospital	DREAMS Subjects [30]
[73]	EEG	1 channel	Wake-NREM-REM	77%	0.56	PSG	16	32–56	Hospital	MIT-BIH [18]
[74]	Pressure sensors	HR + RR + Movement	Wake-REM-Light-Deep	64%	0.46	PSG	66	17–72	Sleep Lab/Home	EarlySense [75]
[76]	EEG	2 channels	Wake-N1-N2-N3-REM	94%	-	PSG	20	25–34	Hospital	Sleep EDF Expanded [32]
[77]	ECG	HRV + EDR	Wake-REM-Light-Deep	75.4%	0.54	PSG	16	32–56	Hospital	MIT-BIH [18]
[78]	PPG + ACC	HRV + Movement	Wake-REM-Light-Deep	49–81%	-	PSG	44 (PLMS)	19–61	Sleep Lab	Fitbit Charge 2 [63]
[79]	ECG	HRV	Wake-REM-Light-Deep	89.20%	-	PSG	3295	-	Home	SHHS Database [55]
[80]	PPG + ACC	HR + Movement	Wake-N1-N2-N3-REM	66.60%	-	PSG	39	19–64	Hospital	Microsoft Band I
[81]	ACC	Movement	Wake-Sleep	85.0%	-	PSG	22	20–45	Home	GT3X+ [36]
[82]	ECG	HRV	Wake-REM-Light-Deep	71.50%	-	PSG	16	32–56	Hospital	MIT-BIH [18]
[83]	ECG	RRI	N3	90%	0.56	PSG	45 (OSA)	-	Hospital	NI DAQ 6221
[84]	ACC	Movement	Wake-Sleep	91%	0.67	PSG	27	18–64	Sleep Lab	myCadian
[85]	ACC	Movement	Wake-Sleep	83%	-	PSG	1817	-	Sleep Lab	MESA Sleep Dataset [86]
[87]	EDA	-	Wake-Sleep	86.0%	-	PSG	91	-	Hospital	Research Device
[88]	ECG	HRV + RRV	Wake-N1-N2-N3-REM	71.16%	0.52	PSG	373 (Various)	22–56	Hospital	SOLAR 3000B
[89]	EEG	1 channel	Wake-S1-S2-S3-S4-REM	93.60%	0.87	PSG	4	-	Hospital	Sleep EDF Expanded [32]
[90]	EEG	1 channel	Wake-N1-N2-N3-REM	81–92%	0.89	PSG	48	20–65	Hospital	Sleep EDF + DREAMS [30,32]
[91]	ACC	Movement	Wake-Sleep	89.65%	-	Sleep Diary	10	-	Home	GT3X [36]
[92]	Pressure sensors	HRV + RRV	Wake-NREM-REM	85%	0.74	PSG	5	63–69	Sleep Lab	Research Device
[93]	EEG	1 channel	Wake-N1-N2-N3-REM	83.50%	-	PSG	20	-	Hospital	Sleep EDF Database [32]
[94]	ECG + ACC + PPG	RRI + HRV + Movement	Wake-REM-Light-Deep	81%	0.69	PSG	32	22–45	Hospital	SensEcho
[95]	PPG + ACC	HR + Movement	Wake-NREM-REM	72%	0.27	PSG	31	19–55	Hospital	Apple Watch (2,3) [96]
[97]	ACC	Movement	Wake-Sleep	85.4%	0.54	EEG	40	18–40	Home	Motion Logger [98]
[99]	PPG + ACC	HRV + RR + Movement	Wake-REM-Light-Deep	77%	0.67	PSG	50	-	-	Samsung Smartwatch [100]
[101]	EEG + EMG	1 channel + chin movement	Wake-N1-N2-N3-REM	81%	-	PSG	49	36–52	Sleep Lab	NTHU Database
[102]	EEG	Ear-EEG	Wake-N1-N2-N3-REM	72.0%	0.59	PSG	15	18–63	Research Lab	Research Device
[103]	PPG + ACC	HR + Movement	Wake-REM-Light-Deep	64%	0.47	PSG	12	19–27	Research Lab	WHOOP strap [104]
[105]	EEG	Ear-EEG	Wake-N1-N2-N3-REM	81%	0.74	PSG	13	18–60	Hospital	Research Device
[106]	Sonar	RR + Movement	Wake-REM-Light-Deep	60%	0.39	PSG	62	31–63	Sleep Lab	Smartphone
[107]	Audio + ACC	RR + HRV + Movement	Wake-REM-Light-Deep	57%	0.36	PSG	53 (OSA)	43–72	Hospital	Research Device
[108]	EEG	Ear-EEG	Wake-N1-N2-N3-REM	74.10%	0.61	PSG	22	19–29	Home	Research Device
[109]	EEG	4 channels	Wake-N1-N2-N3-REM	77.3%	0.69	PSG	243	>18	Hospital	HomePAP Database [110]
[111]	Audio	Breathing/snoring sounds	Wake-NREM-REM	75%	0.42	PSG	13	21–26	Sleep Lab	Research Device
[112]	PPG + ACC	HRV + Movement	Wake-REM-Light-Deep	65–89%	0.54	EEG	35	14–40	Home	Fitbit Charge 2 [63]
[113]	Radar sensors	Respiratory movements	Wake-REM-Light-Deep	62.70%	0.46	PSG	9	23–27	Home	Circadia C100 [114]
[115]	ACC	Movement	Wake-Sleep	90.30%	0.54	PSG	41	>13	Sleep Lab	Arc
[116]	ECG	RRI + RR	Wake-NREM-REM	76.50%	0.49	PSG	25 (SDB)	>18	Hospital	Unknown
[117]	PPG + ACC	HR + Movement	Wake-N1-N2-N3-REM	54.20%	-	PSG	18	-	Sleep Lab	Basis B1
[118]	ACC	Movement	Wake-Sleep	87.70%	0.6	EEG	40	avg. 26.7	Home	Motion Logger [98]
[119]	ACC + PPG	HR + Movement	Wake-Sleep	89.90%	0.42	PSG	8	35–50	Sleep Lab	Apple Watch + Oura [96,120]
[121]	PPG	Oximetry	Wake-N1-N2-N3-REM	64.1%	0.51	PSG	894 (OSA)	44–66	Hospital	Xpod 3011 [122]
[123]	PPG	PPI	Wake-Sleep	81.10%	0.52	PSG	10 (SDB)	43–75	Hospital	Unknown
[124]	ECG + ACC	HRV + Movement	Wake-N1-(N2+N3)-REM	75.9%	0.6	PSG	389 (Multiple)	-	Hospital	SOMNIA Dataset [125]
[126]	ACC	Movement	Wake-Sleep	84.7%	0.45	PSG	43	45–84	Sleep Lab	MESA Sleep Dataset [86]
[127]	ECG	HR	Wake-REM-Light-Deep	77%	0.66	PSG	-	-	Sleep Lab	MESA Sleep + SHHS [55,86]
[128]	ECG + ACC	HRV + Movement	Wake-REM-Light-Deep	66.9%	0.51	PSG	20	20–37	Home	Firstbeat [129]
[130]	ACC	Movement	Wake-Sleep	90.3%	-	PSG	8	18–35	Research Lab	Zulu Watch
[131]	ACC	Finger Movement	Wake-Sleep	85.0%	-	PSG	25	-	Sleep Lab	THIM [132]

PSG—Polysomnography, EEG—Electroencephalography, EOG—Electrooculography, EMG—Electromyography, ECG—Electrocardiography, ACC—Accelerometer, RIP—Respiratory Inductive Plethysmography, PPG—Photoplethysmography, EDA—Electrodermal Activity, REM—Rapid Eye Movement, NREM—Non-Rapid Eye Movement, HBI—Heart Beat Interval, RRI—R-R Interval, PPI—Pulse Peak Interval, HRV—Heart Rate Variability, PRV—Pulse Rate Variability, RRV—Respiratory Rate Variability, HR—Heart Rate, RR—Respiratory Rate, PTT—Pulse Transit Time, EDR—ECG-Derived Respiration, OSA—Obstructive Sleep Apnoea, SDB—Sleep Disordered Breathing, PLMS—Periodic Limb Movements of Sleep.

**Table 2:** Number of articles using each sensing modality as a single data source and in combination with other sensors.

Sensing Modality	As Single Source	In Combination
EEG	19	4
ECG	10	8
EMG	0	2
EOG	0	1
Accelerometer	15	21
RIP	1	3
Radar sensors	4	0
Pressure sensors	4	0
Audio	2	1
PPG	4	16
Nasal airflow	1	1
Sonar sensors	1	0
EDA	1	0

## Abbreviations

The following abbreviations are used in this manuscript:

PSG	Polysomnography
EEG	Electroencephalography/Electroencephalogram
EOG	Electrooculography/Electrooculogram
EMG	Electromyography/Electromyogram

ECG	Electrocardiography/Electrocardiogram
BCG	Ballistocardiography/Ballistocardiogram
ACC	Accelerometer
RIP	Respiratory Inductive Plethysmography
EDA	Electrodermal Activity
PPG	Photoplethysmography/Photoplethysmogram
REM	Rapid Eye Movement
NREM	Non-Rapid Eye Movement
R&K	Rechtschaffen and Kales
AASM	American Academy of Sleep Medicine
HBI	Heart Beat Interval
RRI	R-R Interval
PPI	Pulse Peak Interval
HRV	Heart Rate Variability
PRV	Pulse Rate Variability
RRV	Respiratory Rate Variability
HR	Heart Rate
RR	Respiratory Rate
PTT	Pulse Transit Time
EDR	ECG-Derived Respiration
LED	Light Emitting Diode
IoT	Internet of Things
OSA	Obstructive Sleep Apnoea
SDB	Sleep Disordered Breathing
PLMS	Periodic Limb Movements of Sleep
